# The Causal Effect of Gut Microbiota and Plasma Metabolome on Lung Cancer and the Heterogeneity across Subtypes: A Mendelian Randomization Study

**DOI:** 10.3390/jpm14050453

**Published:** 2024-04-25

**Authors:** Jun Zeng, Bin Yi, Ruimin Chang, Jiashuo Li, Jiebo Zhu, Zhongjie Yu, Xi Li, Yang Gao

**Affiliations:** 1 Department of Thoracic Surgery, Xiangya Hospital, Central South University, Changsha 410008, China; drzengjun@163.com (J.Z.); yibin0529@csu.edu.cn (B.Y.); changruimin@csu.edu.cn (R.C.); ljs1001@csu.edu.cn (J.L.); zhujiebo97@163.com (J.Z.); 228112297@csu.edu.cn (Z.Y.); 2 Hunan Engineering Research Center for Pulmonary Nodules Precise Diagnosis & Treatment, Changsha 410008, China; 3 National Clinical Research Center for Geriatric Disorders, Changsha 410008, China; 4 Xiangya Lung Cancer Center, Xiangya Hospital, Central South University, Changsha 410008, China; 5 Departments of Clinical Pharmacology and Respiratory Medicine, Hunan Key Laboratory of Pharmacogenetics, and National Clinical Research Center for Geriatric Disorders, Xiangya Hospital, Central South University, Changsha 410008, China; 6 Institute of Clinical Pharmacology, Engineering Research Center for Applied Technology of Pharmacogenomics of Ministry of Education, Central South University, Changsha 410008, China

**Keywords:** lung cancer, gut microbiota, plasma metabolome, mendelian randomization, biomarker

## Abstract

The causal effect and pathways of gut microbiota and plasma metabolome on lung cancer have been important topics for personalized medicine; however, the heterogeneity of lung cancer subtypes has not gained enough attention in previous studies. This study sought to employ a Mendelian randomization analysis to screen the specific gut microbiota and plasma metabolome, which may have a causal effect on lung cancer. We further extended our analysis to estimate the effects of these exposures on various pathological subtypes of lung cancer. Furthermore, a mediation analysis was performed to identify the potential pathway underlying the influence of microbiota and metabolites. Our study identified 13 taxa and 15 metabolites with a causal association with the overall risk of lung cancer. Furthermore, we found 8 taxa and 14 plasma metabolites with a causal effect on lung adenocarcinoma, 4 taxa and 10 metabolites with a causal effect on squamous cell lung carcinoma, and 7 taxa and 16 metabolites with a causal effect on SCLC. We also identified seven mediation pathways that could potentially elucidate the influence of these microbiota and metabolites on overall lung cancer or special subtypes. Our study highlighted the heterogeneity of the gut microbiome and plasma metabolome in a lung cancer subtype and elucidated the potential underlying mechanisms. This could pave the way for more personalized lung cancer prevention and treatment.

## 1. Introduction

Lung cancer (LC) is a devastating disease with the highest incidence and mortality among all malignancies worldwide [1]. Lung adenocarcinoma (LUAD), squamous cell lung cancer (SCLC), and small cell lung cancer (SCC) are the most common pathological subtypes of LC, accounting for more than 90% of all LC cases [2]. Each subtype has distinct morphological features, molecular profiles, and clinical behaviors that impact their diagnosis, prognosis, and treatment strategies [3]. Understanding the heterogeneity of pathogenesis across these subtypes is essential for developing precise therapies and personalized treatment strategies for patients. Epidemiological studies have hinted at the heterogeneity of pathogenesis across LC pathological subtypes [4]. For example, SCLC is most common in male smokers, while LUAD is generally thought to be related to genetic and environmental factors. Moreover, distinct molecular alterations have been found to drive the development and progression of different subtypes of lung cancer. For example, LUAD commonly exhibits driver mutations in genes such as EGFR, KRAS, and EML4-ALK, allowing for targeted therapy with tyrosine kinase inhibitors. In contrast, SCLC is characterized by TP53 and RB1 mutations, making it aggressive and initially responsive to chemotherapy and radiation but prone to rapid resistance [5]. SCC, on the other hand, often arises from chronic inflammation and tobacco exposure, leading to frequent TP53 mutations and chromosomal instability. While SCC treatment typically involves surgery, radiation, and chemotherapy, targeted therapies have been less successful compared to LUAD [6,7]. An increasing number of studies have unveiled subtype-specific biomarkers and therapeutic targets, emphasizing the importance of personalized treatments tailored to each patient’s tumor characteristics [8].

In recent years, several studies showed the important role of gut microbiota (GM) in LC pathogenesis and treatment. The results from Lu et al. showed that Ruminococcus gnavus was significantly upregulated in LC patients, with Firmicutes, Clostridia, Bacteroidacea, Bacteroides, and Lachnospira being enriched in the healthy population [9]. Lee et al. showed that bifidobacterium could improve sensitivity to immune checkpoint inhibitors (ICIs) in non-small cell lung cancer (NSCLC) patients [10]. By analyzing stool samples from sixteen NSCLC patients treated with ICIs, Huang et al. reported an overrepresentation of parabacteroides distasonis, bacterium LF-3, sutterella wadsworthensis HGA0223, and bacteroides vulgatus in patients with a favorable response to ICIs [11]. Derosa et al. suggested that GM might mediate the negative association between antibiotics and ICIs activity in patients with advanced NSCLC and renal cell cancer [12]. 

The plasma metabolome (PM) is thought to be an important medium for GM. For colorectal cancer, lactobacillus gallinarum-derived indole-3-lactic acid could protect against intestinal tumorigenesis by promoting the apoptosis of colorectal cancer cells [13]. For LUAD, PM, and GM were also found to be associated with cancer invasive grades. Zhao et al. revealed the serum-level differences in glycerophospholipids, imidazopyrimidines, and AcylGlcADG 66:18 between LC patients and healthy volunteers. Furthermore, they showed that LC microbes were associated with metabolites, such as Erysipelotrichaceae_UCG_003, Clostridium, and Synergistes, which were associated with glycerophospholipids [14]. However, traditional etiology research struggled to establish causality and the heterogeneity did not gain enough attention in previous studies to reveal the effect of GM and PM on LC. The causal effect of GM and PM on LC and the heterogeneity across subtypes remain controversial.

Mendelian randomization (MR) is a method in epidemiology that uses single-nucleotide polymorphisms (SNPs) as instrumental variables (IVs) to evaluate the causal effect of exposure on outcome [15]. This method minimizes two major concerns with other observational epidemiologic study designs. Due to the inherently random allocation, genetic variants are independent of confounders, which enables the inference of causal effects in the presence of unobserved confounding. Moreover, MR results are protected against reverse causation bias as genetic variants are certainly randomly allocated before the progression of disease [16].

There are several advantages of MR [17]. Firstly, it could address many limitations of observational study design, including confounding, reverse causation, and the demonstration of causality. Secondly, compared to conducting a randomized clinical trial, MR requires less time and expense. Thirdly, MR allows for an analysis of existing studies. Fourthly, MR could address questions that randomized clinical trials were unable to answer.

To evaluate the causal role of GM and PM across LC subtypes, we integrated a systematic MR, utilizing previously published genome-wide association study (GWAS) summary data.

## 2. Materials and Methods

### 2.1. Study Design and Data Sources

This study followed the STROBE-MR guidelines for reporting MR results [18]. The flowchart is depicted in Figure S1. We obtained summary GWAS databases for LC, LUAD, SCC, and SCLC from the GWAS Catalog. The databases GCST004744, GCST004748, GCST004746, and GCST004750 were sourced from a meta-analysis which investigated traits related to LC with a large sample size and minimal sample overlap [19]. In the plasma metabolites database, a total of 452 plasma metabolites were examined in a population of 7824 Europeans [20]. Additionally, the GM GWAS results were sourced from the MiBioGen program. The MiBioGen program was a large-scale international research project which evaluated the associations between the abundance of 211 gut taxa and whole-genome SNPs in over 18,000 individuals from more than 20 labs worldwide. In this program, 16r DNA sequencing was utilized to analyze taxa abundance, and genotyping information was evaluated using whole-genome genotyping microarrays [21]. The details of GWAS databases included in this study are summarized in Table S1. 

### 2.2. Instrumental Variables Screening and Mendelian Randomization Analysis

During the IVs for the GM and PM screening process, we dropped all palindromic SNPs from the analysis to ensure we could comprehensively capture the relevant information. To ascertain the causal relationship between GM and LC, or PM and LC, we employed different methods of MR based on the number of IVs. If there were at least two IVs available, the Inverse Variance Weighted (IVW) method was performed. In cases where only one or two IVs were present, the Wald ratio model was applied. To investigate the heterogeneity and pleiotropy of the selected exposure, we employed MR–Egger’s intercept and the Cochran Q test. Only exposure that exhibited no heterogeneity or pleiotropy (with a pleiotropy *p*-value > 0.05 and a heterogeneity Q-value > 0.05) was included in subsequent analyses. We performed an MR Steiger test of directionality. The strength of the IVs was assessed by calculating the F-statistic. If the corresponding F-statistic was <10, it was considered that there was significant weak instrumental bias and the IV was dropped from this study. This rigorous screening process ensured that our analysis was based on robust and reliable exposure, thereby enhancing the validity of our findings.

### 2.3. Enrichment Analysis

For metabolites identified to have a causal effect on LC, we performed an enrichment analysis based on the Kyoto Encyclopedia of Genes and Genomes (KEGG) and Small Molecule Pathway Database (SMDPB) to explore the potential functions of this metabolism, including metabolite pathway enrichment analysis and pathway activity profilin. The enrichment analysis was conducted by MetaboAnalyst 5.0 (https://www.metaboanalyst.ca/, accessed on 19 August 2023).

### 2.4. Mediation Analysis

Mediation analysis was a method used to evaluate the role of a third variable in the mechanisms of exposure-induced outcomes. Through mediation MR, we identified a pathway from GM to PM to LC, which helps elucidate the potential mechanism by which GM could contribute to LC. Initially, we performed a mediation analysis to evaluate the possible link between GM and PM identified by MR. Subsequently, we evaluated the ‘indirect’ effect of GM on LC through a two-step MR process. The proportion of the mediation effect of the PM was calculated using the following formula [22,23]:

$$PM=\frac{\beta GP\;*\;\beta PL}{\beta GL}$$

In this formula, PM represents the mediation effect of metabolites, βGP represents the causal MR effect of GM on PM, βML represents the MR causal effect of PM on LC, and βGL represents the ‘total’ effect of GM on LC.

### 2.5. Software and Pre-Registration

We used R (version 4.1.3) package TwoSampleMR (version 0.4.26) to conduct IV selection, as well as Mendelian randomization and mediation analysis [24]. The flowchart was produced by Fig-draw (https://www.figdraw.com/ accessed on 21 November 2023).

## 3. Results

### 3.1. Gut Microbiome Mendelian Randomization Analysis

**Figure 1:**
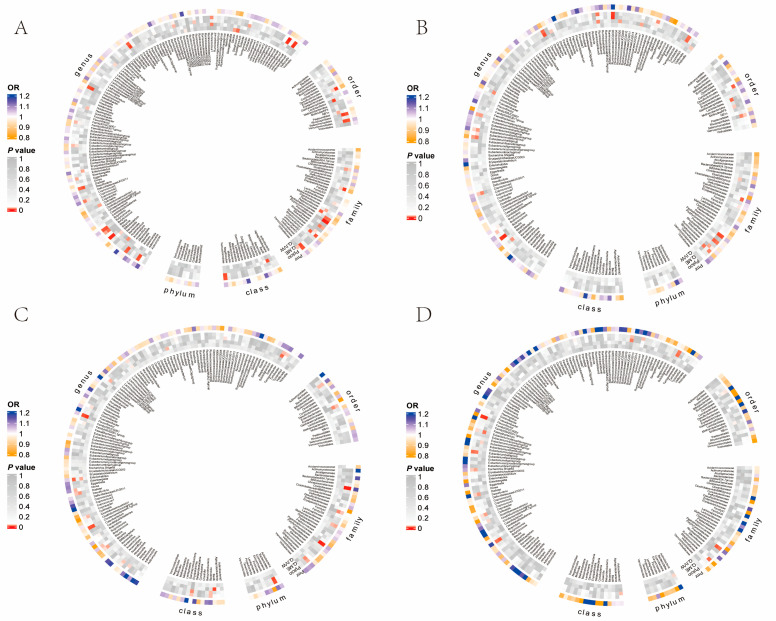
Results of a Mendelian randomization analysis and sensitivity analysis between gut microbiota and lung cancer (**A**), lung adenocarcinoma (**B**), squamous cell carcinoma (**C**), and small cell lung cancer (**D**) (locus-wide significance, *p* < 1 × 10^−5^).

**Figure 2:**
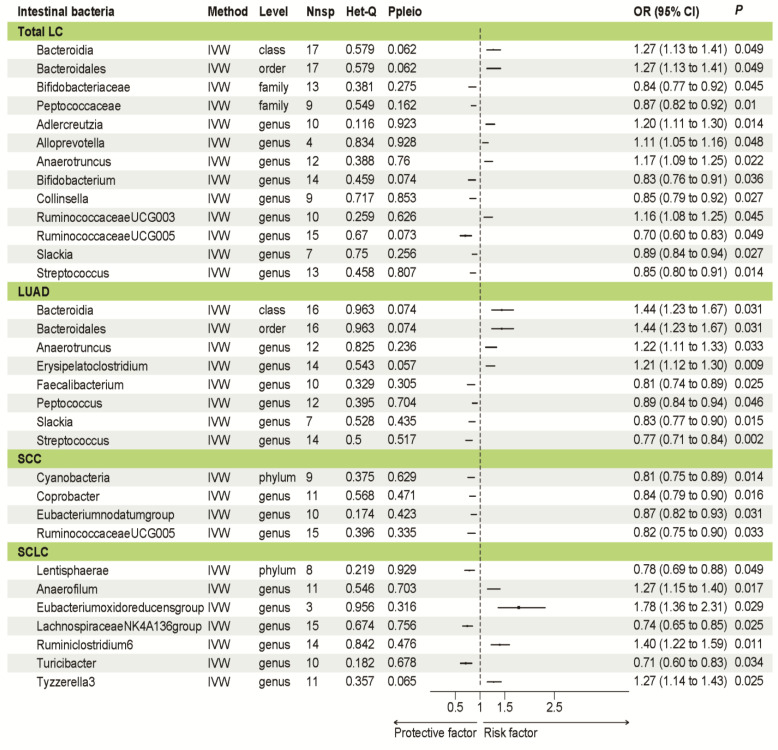
Mendelian randomization results of gut taxa with a causal relationship to outcomes. Abbreviation: LC, lung cancer; LUAD, lung adenocarcinoma; SCC, squamous cell carcinoma; SCLC, small cell lung cancer; Nsnp, number of single-nucleotide polymorphisms; IVW, inverse variance weighted; OR, odds ratio.

Through univariate MR analysis, we examined the causal effect of all gut taxa on LC (Figure 1A), LUAD (Figure 1B), SCC (Figure 1C), and SCLC (Figure 1D). We found six taxa which exhibited a positive causal effect on LC and seven taxa which showed a protective causal effect. When analyzing according to histopathological subtype, we identified eight taxa with a significant causal effect on LUAD (including four taxa that exhibited a positive causal effect and four taxa that showed a protective causal effect), four taxa with a significant positive causal effect on SCC, and seven taxa with a significant causal effect on SCLC (including four taxa that exhibited a positive causal effect and three taxa that showed a protective causal effect) (Figure 2). In exploring the taxa shared between subgroups, we found five taxa shared by LUAD and LC, and one taxon shared by SCC and LC (Figure S2). Slackia and streptococcus showed a positive association with LC and LUAD risk. RuminococcaceaeUCG005 was a risk factor associated with SCC and LC risk. Notably, no taxon was shared by different pathological subtypes, suggesting that the effect of GM varies across different pathological subtypes of LC. This finding underscored the heterogeneity of the relationship between GM and LC.

### 3.2. Plasma Metabolome Mendelian Randomization Analysis

**Figure 3:**
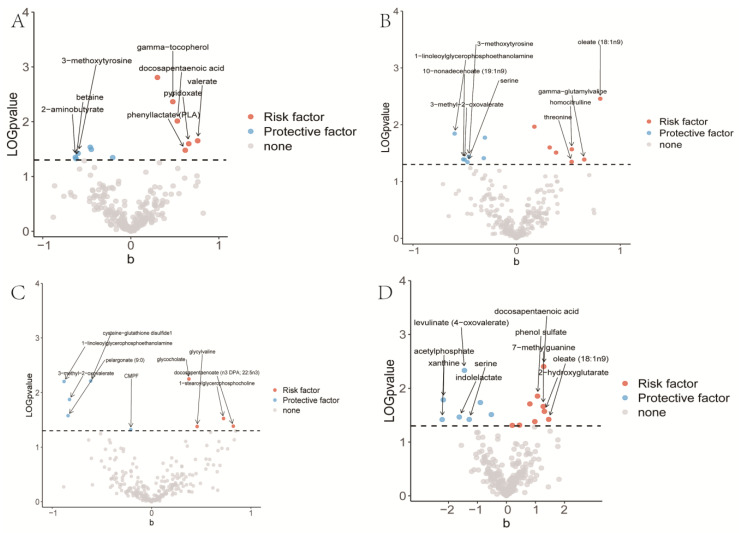
Results of Mendelian randomization analysis between plasma metabolome and lung cancer (**A**), lung adenocarcinoma (**B**), squamous cell carcinoma (**C**), and small cell lung cancer (**D**).

**Figure 4:**
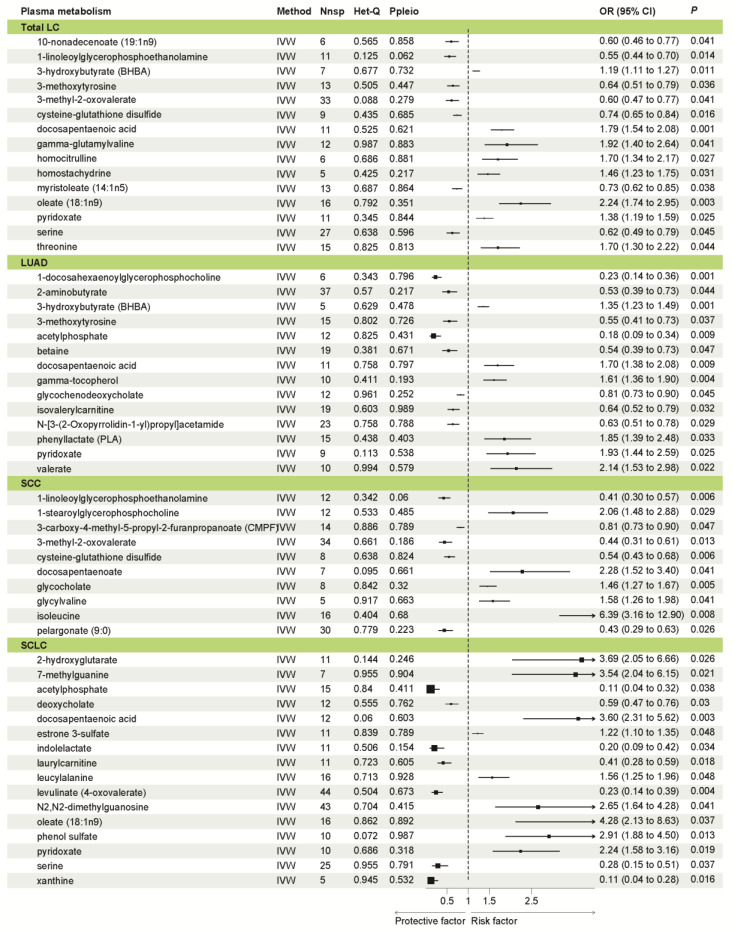
Mendelian randomization results of plasma metabolome with a causal relationship to outcomes. Abbreviation: LC, lung cancer; LUAD, lung adenocarcinoma; SCC, squamous cell carcinoma; SCLC, small cell lung cancer; Nsnp, number of single-nucleotide polymorphisms; IVW, inverse variance weighted; OR, odds ratio.

As shown in Figure 3, we conducted an analysis of the causal effect of 452 plasma metabolites on LC, LUAD, SCC, and SCLC. In total, we identified fifteen plasma metabolites showed a causal association with LC, including eight metabolites with a positive causal effect and seven metabolites with a protective causal effect. In LUAD, we found fourteen plasma metabolites with a causal effect, comprising eight metabolites with a protective effect and six metabolites with a positive effect. In SCC, we found an equal number of protective and risk metabolites, totaling 10. For SCLC, we identified a total of sixteen metabolites with significant causal effect, including seven protective and nine risk metabolites (Figure 4). Interestingly, there were some factors shared by subtypes of LC. For instance, Docosapentaenoic acid (DPA) was a risk factor shared by total LC, LUAD, SCC, and SCLC (Figure S3). These results showed the causal effect of PM on LC and the heterogeneity of PM across LC subtypes.

### 3.3. Enrichment Analysis

**Figure 5:**
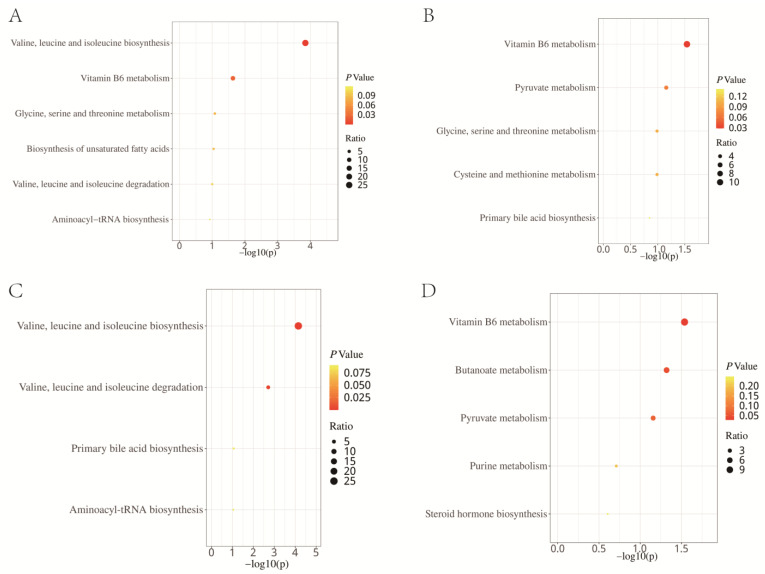
Enrichment analysis results of the causal metabolites of lung cancer (**A**), lung adenocarcinoma (**B**), squamous cell carcinoma (**C**), and small cell lung cancer (**D**) based on the Kyoto Encyclopedia of Genes and Genomes database.

**Figure 6:**
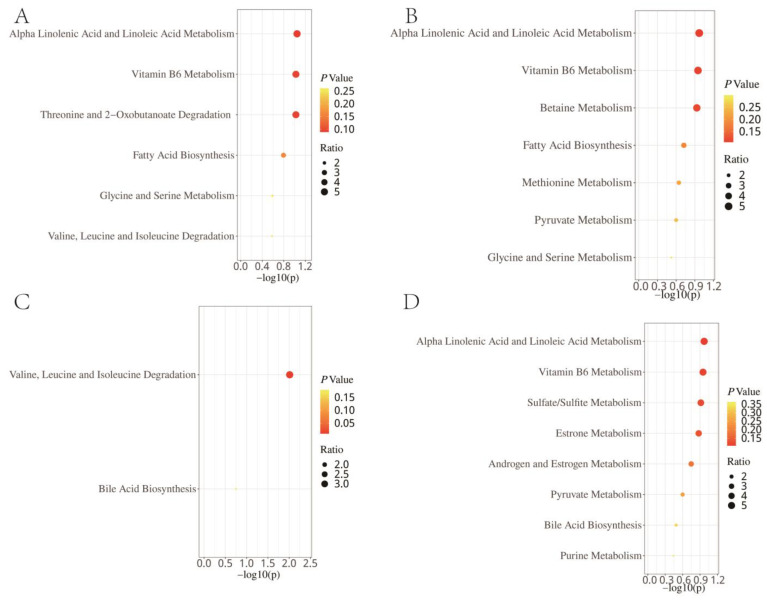
Enrichment analysis results of the causal metabolites of lung cancer (**A**), lung adenocarcinoma (**B**), squamous cell carcinoma (**C**), and small cell lung cancer (**D**) based on the Small Molecule Pathway Database.

Enrichment analysis, based on the KEGG, revealed that the 15 metabolites with a causal effect on total LC were significantly enriched in the pathways of valine, leucine, and isoleucine biosynthesis, as well as the vitamin B6 metabolism (Figure 5A). In the SMPDB, these metabolites were enriched in the alpha-linolenic acid and linoleic acid metabolism, the vitamin B6 metabolism, and threonine and 2-oxobutanoate degradation (Figure 6A). When we conducted an enrichment analysis based on pathological subtype, we found that the metabolites with a causal effect on LUAD were enriched in the vitamin B6 metabolism pathway according to KEGG (Figure 5B), and in the alpha-linolenic acid and linoleic acid metabolism, vitamin B6 metabolism, and betaine metabolism according to SMPDB (Figure 6B). For SCC, 10 metabolites were enriched in valine, leucine, and isoleucine biosynthesis in both KEGG (Figure 5C) and SMPDB (Figure 6C). For SCLC, KEGG-based results showed that a total of 16 metabolites were enriched in the vitamin B6 metabolism, butanoate metabolism, and pyruvate metabolism (Figure 5D). The SMPDB-based results showed that these 16 metabolites were enriched in the alpha-linolenic acid and linoleic acid metabolism, vitamin B6 metabolism, sulfate/sulfite metabolism, and estrone metabolism (Figure 6D).

### 3.4. Mediation Analysis Results

**Figure 7:**
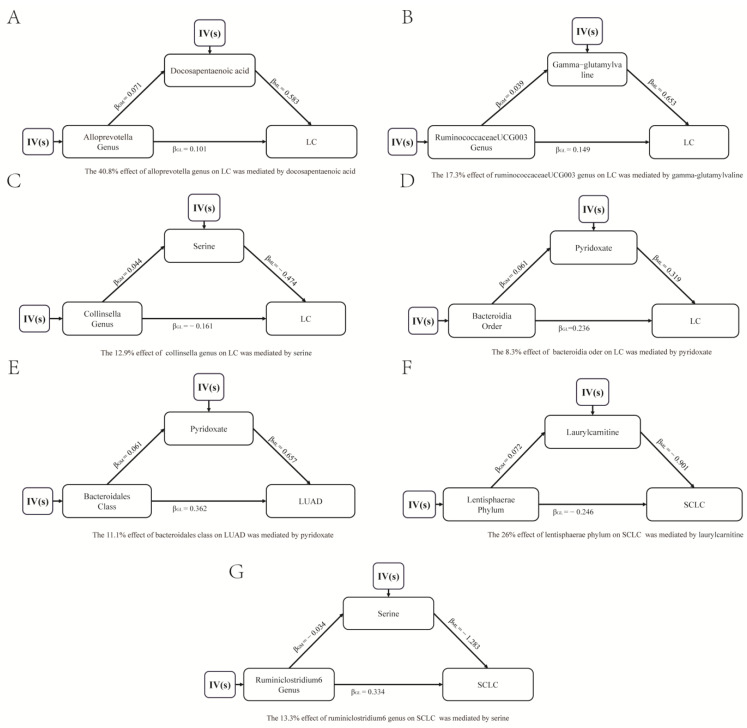
Mediation effect relationship of bacteria and metabolites on lung cancer (**A**–**D**), lung adenocarcinoma (**E**), and small cell lung cancer (**F**,**G**).

The taxa and metabolites with a significant causal effect on LC in MR analysis were included in mediation analysis to identify the potential pathways through which the GM and PMs affect LC. This analysis revealed a total of four mediation pathways: 40.8% of the effect of the alloprevotella genus on LC was mediated by DPA (Figure 7A); 17.3% of the effect of the ruminococcaceae UCG-003 genus on LC was mediated by gamma-glutamylvaline (Figure 7B); 12.9% of the effect of the collinsella genus on LC was mediated by serine (Figure 7C); 8.3% of the effect of the bacteroidia order on LC was mediated by pyridoxate (Figure 7D). In LUAD, 11.1% of the effect of the bacteroidales class on LUAD was mediated by pyridoxate (Figure 7E), In SCLC, two significant mediation pathways were identified: 13.3% of the effect of the ruminiclostridium 6 genus on SCLC was mediated by Serine (Figure 7F); 26% of the effect of the lentisphaerae phylum on SCLC was mediated by laurylcarnitine (Figure 7G).

## 4. Discussion

In this study, we explored the causal relationship between GM, PMs, and LC. We identified 13 taxa and 15 metabolites which were causally associated with LC risk. These metabolites were predominantly enriched in the biosynthesis of valine, leucine, and isoleucine, as well as the vitamin B6 metabolism. Through a mediation analysis of these exposures with a significant causal effect on LC, we identified four pathways. When conducting an analysis based on pathological subtypes, we discovered eight taxa and fourteen plasma metabolites with a causal effect on LUAD, four taxa and ten metabolites with a causal effect on SCC, and seven taxa and sixteen metabolites with a causal effect on SCLC. We further investigated the shared factors between different subtypes. Among GM, no taxon was shared by different subtypes. However, among PMs, DPA was common to LUAD, SCC, and SCLC. The enrichment in each subgroup demonstrated tumor heterogeneity. For LUAD, these metabolomes were enriched in the vitamin B6 metabolism. Conversely, for SCC, these metabolomes were enriched in valine, leucine, and isoleucine biosynthesis. In the mediation analysis, we did not find any pathway shared by LUAD, SCC, and SCLC.

In this study, MR was utilized to assess the causal effect of GM and PMs on LC and the major subtypes. Previous epidemiological research has identified various risk factors associated with LC, including long-term smoking, genetic predisposition, environmental pollution, occupational exposures, and contact with radioactive substances [25]. However, these studies had limitations in establishing a definitive causal relationship. Unlike traditional epidemiological studies, MR utilized genetic variations present at birth to estimate causal relationships between risk factors and health outcomes, resembling a randomized controlled trial design. SNPs are independent of lifestyle and environmental factors, which served as IVs in MR analyses, isolating direct causal effects. Therefore, MR could mimic randomized controlled trials, reducing potential confounders and issues of reverse causality [26]. This method was valuable in uncovering the biological mechanisms of complex diseases when traditional observational studies are limited or unethical. By implementing this causal inference model, our study rigorously examined whether alterations in GM and PMs had a causal effect on LC, rather than just being correlated with it. This approach allowed a more confident determination of the causal role of GM and PMs in the pathogenesis of LC.

The GM had effect on various metabolic pathways, including the lipid metabolism and endogenous vitamin synthesis, making PMs a significant mediator of GM in the body [27,28]. The metabolites produced by the GM can travel to different parts of the body through the gut–brain axis, gut–lung axis, and gut–hepatic axis, potentially disrupting the body’s physiological balance [29,30,31,32]. Research by Ji Ma et al. demonstrated that GM was linked to reduced proliferation and invasion, as well as increased apoptosis, in breast cancer cells [33]. Another study by Su et al. revealed that lactobacillus can stimulate the production of IL-35+ B cells by generating 3-idoleacetic acid when exposed to lipopolysaccharide [34]. Our study identified 15 metabolites with a causal relationship to lung cancer (LC), with DPA showing the strongest correlation. DPA, an omega-3 polyunsaturated fatty acid found in fish and lean red meat, was known for its anti-inflammatory and anti-tumor properties [35]. However, previous research by Liu et al. suggested a higher risk of LC, LUAD, and SCC associated with DPA [36]. Our findings supported this link and highlighted DPA as a key mediator in the impact of alloprevotella on LC. Alloprevotella has been consistently found at higher levels in the tumor mucosa compared to normal mucosa and feces [37,38]. Our study further elucidated the pathogenic mechanism by which alloprevotella contributes to LC though DPA.

Through an enrichment analysis based on the KEGG database, we discovered that the metabolites with significant causal effects on LC were enriched in valine, leucine, isoleucine biosynthesis, and vitamin B6 metabolism pathways. An analysis based on the SMPDB database revealed these metabolites were enriched in the alpha-linolenic acid and linoleic acid metabolism, the vitamin B6 metabolism, and threonine and 2-oxobutanoate degradation. Previous studies have shown that alterations in the vitamin B6 metabolism were associated with multiple diseases. The proficiency of the vitamin B6 metabolism could modulate the adaptive response of tumor cells to various physical and chemical stress conditions and was a good prognostic marker in NSCLC patients [39,40,41]. However, it should be noted that using individual vitamin B6 supplements was associated with a 30 percent to 40 percent increase in LC risk among men [42]. Our results showed that PMs might increase the risk of LC through the vitamin B6 metabolism.

The GM’s crucial role in intestinal function has led to increased interest in its potential impact on cancer. Cao et al. found that gut microbiome shifts were associated with colorectal cancer-associated T cell receptor repertoire abnormalities [43]. In a stage I prospective clinical trial, Bertrand et al. demonstrated that fecal microbiota transplantation from healthy donors plus ICIs was safe for patients with advanced melanoma [44]. In this study, seven potential pathways were identified to explain how PM mediated the impact of GM on LC. The results revealed the molecular mechanisms through which GM influenced the development of LC and provided a potential target for improving the therapeutic effect. For example, serine was found to play a protective role in LC and mediated 12.9% of the effect of the collinsella genus. In a previous study, serine was known to play a crucial role in anti-tumor immunity regulation, having an influence on both tumor pathogenesis and immunotherapy efficacy [45]. Additionally, collinsella has been observed to be enriched in patients who respond well to immunotherapy [46]. Therefore, this study not only enhanced understanding of the pathological mechanisms underlying LC but also laid the groundwork for future intervention strategies targeting GM and PMs. This is a promising way to improve LC prevention, diagnosis, and treatment outcomes by modulating the microbiota composition of patients.

Traditionally, LUAD, SCC, and SCLC were categorized under LC. However, recent advancements and growing evidence suggest that these should be viewed as distinct cancers with unique characteristics [47]. SCLC is known for its aggressive nature, rapid growth, and high metastatic potential, with a 5-year survival rate of only 6.1%. In contrast, LUAD and SCC generally achieve better prognoses, with LUAD showing 5-year survival rates of 34–52%, indicating a less aggressive course [48]. Treatment responses also vary among these subtypes, with LUAD being more responsive to targeted therapies due to its molecular profile, while SCC and SCLC show better responses to traditional chemotherapy and emerging immunotherapies [49]. Our detailed analysis of tissue samples revealed differences in the underlying pathogenesis of LUAD, SCC, and SCLC, emphasizing the importance of understanding the distinct biological pathways involved in each subtype for effective treatment strategies. The enrichment analysis highlighted the varying sensitivities of these cancer types to different metabolic pathways. Interestingly, both LC and SCLC demonstrated increased sensitivity to disruptions in the vitamin B6 metabolism, suggesting potential new therapeutic options. Conversely, SCC showed a stronger response to changes in the biosynthesis pathway of valine, leucine, and isoleucine, essential amino acids that are crucial for cellular metabolism and growth. These findings emphasize the necessity of personalized medicine approaches that consider the specific metabolic needs of individual lung cancer subtypes, leading to more tailored and effective treatments.

This study has significant clinical implications. Firstly, utilizing MR, we successfully identified the causal effect of GM and PMs on LC. This discovery provides reliable biomarkers for early detection and preventive measures against LC. By pinpointing these specific microbial changes, clinicians could potentially intervene before the onset or progression of the disease, significantly improving patient outcomes. Secondly, this research explored the intricate mechanistic pathways through which GM influences LC by modulating PMs. The results enhance the understanding of how the GM contributes to the progression of LC and provide potential therapeutic targets. Lastly, we explored the heterogeneity of GM and PMs across SCC, LUAD, and SCLC. The results highlighted the distinct variations in their respective pathogeneses, emphasizing the critical importance of considering the pathological subtype when diagnosing and devising treatment strategies for LC patients. This could lead to more personalized medicine.

There were inevitably also several limitations in our study. Firstly, our shallow GM database only provided taxonomic classification up to the genus level. This limitation hindered our ability to identify specific species-level taxa that could directly influence LC, preventing us from drawing definitive conclusions about the microbial agents involved in LC pathogenesis or prevention. Additionally, despite our efforts to reduce biases using statistical techniques like MR–Egger’s intercept and the Cochran Q test, analyzing the summarized data could still introduce bias. While these methods helped us to address unmeasured confounding factors, the use of summary statistics lacks the precision and control of individual-level data, potentially impacting the accuracy of results. Another significant limitation was the demographic composition of our dataset, primarily comprising individuals of European descent. This homogeneity raises concerns about population stratification and limits the generalizability of our findings to other ethnicities and populations beyond Europe. The presence of population-specific variations in gut microbiota profiles and disease susceptibility emphasizes the need for caution when interpreting our results across a diverse population.

## 5. Conclusions

This comprehensive MR study provides evidence supporting the causal relationship between GM and PMs in the progression of LC and its major subtypes, including LUAD, SCC, and SCLC. The study also reveals various pathways through which PMs mediate the impact of GM on LC, emphasizing the intricate connection between gut health and LC. Furthermore, the research showed the heterogeneity in both GM and PMs across different LC subtypes, suggesting potential distinct mechanisms through which these microbiomes can affect the development and characteristics of specific LC subtypes. The identification of specific GM and associated PMs could serve as a valuable resource for identifying therapeutic targets and advancing personalized medicine interventions.

## Data Availability

The primary data included in this study were derived from public databases and the download addresses could be found in Table S1.

## Supplementary Materials

The following supporting information can be downloaded at: https://www.mdpi.com/article/10.3390/jpm14050453/s1, Figure S1: Research flowchart; Figure S2: The vein plot of gut microbiota shared by lung cancer, lung adenocarcinoma, squamous cell carcinoma, and small cell lung cancer; Figure S3: The vein plot of plasma metabolome shared by lung cancer, lung adenocarcinoma, squamous cell carcinoma, and small cell lung cancer; Table S1: the detail of database included in this study.
