# Supplementary material for: The Causal Effect of Gut Microbiota and Plasma Metabolome on Lung Cancer and the Heterogeneity across Subtypes: A Mendelian Randomization Study
Source: J Pers Med. 2024 Apr 25;14(5):453. doi: 10.3390/jpm14050453 (PMC11122438; doi:10.3390/jpm14050453)
Supplement: Supplementary file 1 [file jpm-14-00453-s001.zip › jpm-2929244-supplementary.pdf]

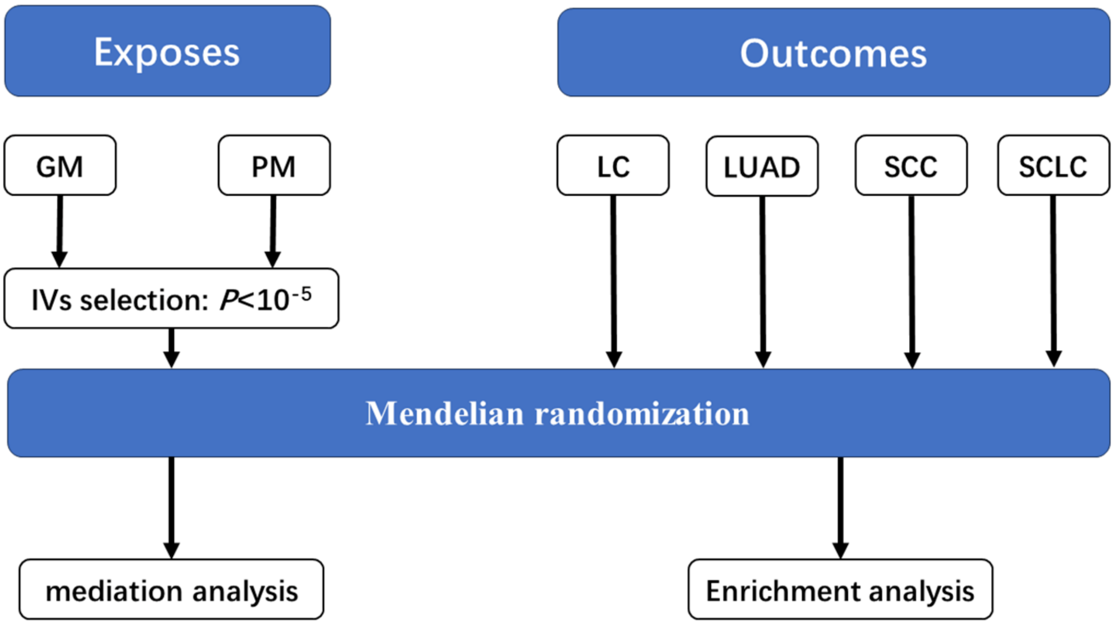

Figure S1. Research flowchart. Abbreviation: GM, Gut microbiota; PM, Plasma metabolome; IVs, Instrumental variables; LC, Lung cancer; LUAD, Lung adenocarcinoma; SCC, Squamous cell carcinoma; SCLC, Small cell lung cancer

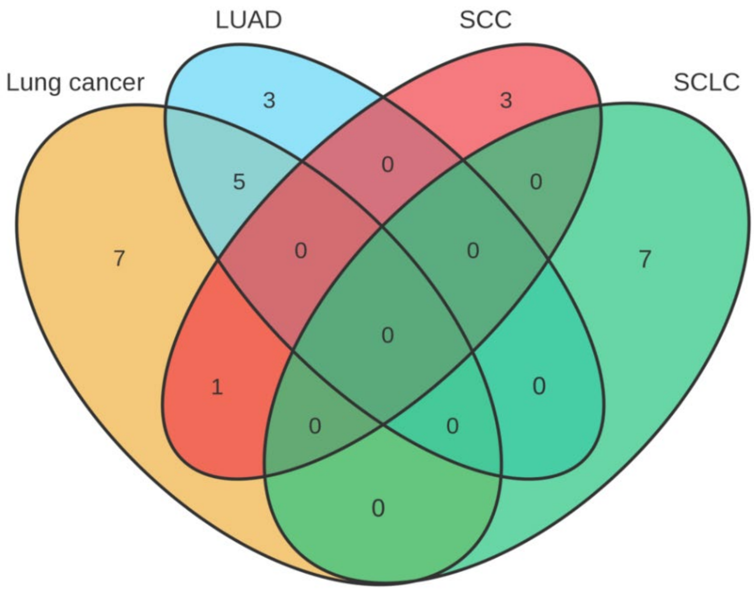

Figure S2. The vein plot of gut microbiota shared by lung cancer, lung adenocarcinoma, squamous cell carcinoma, and small cell lung cancer. Abbreviation: LC, Lung cancer; LUAD, Lung adenocarcinoma; SCC, Squamous cell carcinoma; SCLC, Small cell lung cancer

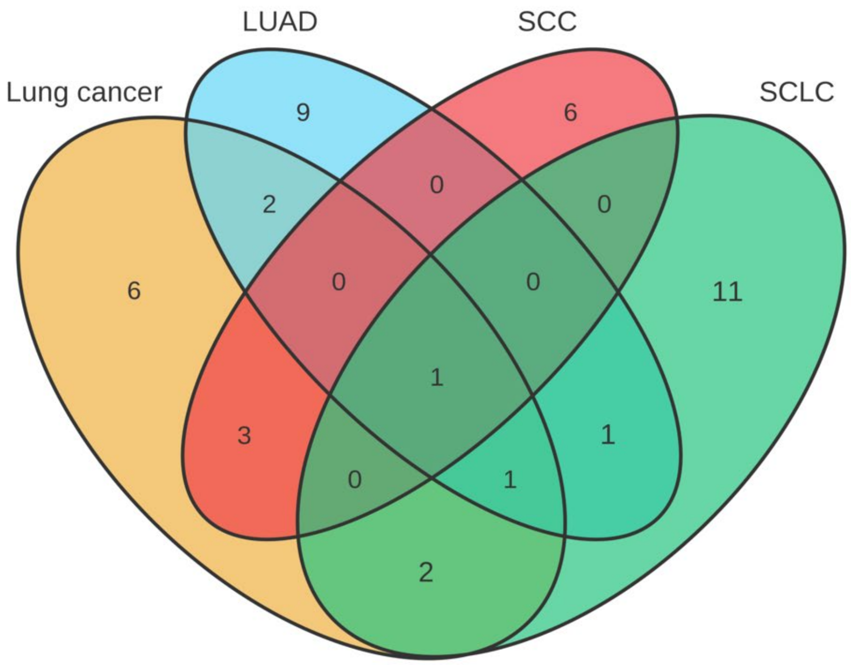

Figure S3. The vein plot of plasma metabolome shared by lung cancer, lung adenocarcinoma, squamous cell carcinoma, and small cell lung cancer. Abbreviation: LC, Lung cancer; LUAD, Lung adenocarcinoma; SCC, Squamous cell carcinoma; SCLC, Small cell lung cancer

Table S1. The detail of database included in this study

Variable	First author	Data ID	Years	Case	Contral	Population
LC	McKay JD	GCST004748	2017	29,266	56,450	100% European
LUAD	McKay JD	GCST004744	2017	11,273	55,483	100% European
SCC	McKay JD	GCST004750	2017	7,426	55,627	100% European
SCLC	McKay JD	GCST004746	2017	2664	21444	100% European
PM	Shin	met-a-459	2014	7703	-	100% European
GM	Alexander Kurilshikov	MIbiogen	2021	18340	-	Mixed

Abbreviation: LC, lung cancer; LUAD, Lung adenocarcinoma; SCC, Squamous cell lung carcinoma; SCLC, Small cell lung cancer; PM, Plasma metabolites; GM, Gut microbiota
